# Sorting out the cell biology of Alzheimer’s disease: focus on BACE1 and APP

**DOI:** 10.1186/1750-1326-7-S1-L6

**Published:** 2012-02-07

**Authors:** Wim Annaert

**Affiliations:** 1 Department of Human Genetics, Katholieke Universiteit Leuven, Belgium

## Background

Amyloid β (Aβ) peptides, the primary constituents of senile plaques and a hallmark in Alzheimer’s disease pathology, are generated through the sequential cleavage of amyloid precursor protein (APP) by BACE1 and γ-secretase. Evidence is accumulating that the early endosome constitutes a major compartment for APP processing; however, the mechanisms of how BACE1 encounters APP are largely unknown.

## Methods

HeLa cells are widely used as cellular models to unravel transport itineraries as they are extremely well characterized and suited for both biochemical and imaging studies. Major findings are extrapolated to primary neurons to demonstrate that newly revealed regulatory mechanisms are conserved in neurons and hence relevant in the light of neurodegeneration. Studies are performed on endogenous proteins as well as through overexpression using either wt or fluorescently-tagged constructs in combination with dominant mutants of small GTPases to block selective transport routes.

## Results

Co-expression of either BACE1 or APP with RAB5-Q79L, a GTP-locked RAB5 mutant, results in their entrapment in enlarged endosomes. β-Cleaved ectodomain fragments of APP accumulate in the lumen of these endosomes, as this accumulation could be prevented by BACE1 inhibitors or is absent in BACE1-/- fibroblasts transfected with RAB5-Q79L. Blocking clathrin-mediated internalization using siRNA against AP-2 complexes or mutant AP180, affected strongly APP endocytosis while BACE1 internalization persisted. Hence, in contrast to APP internalization which is clathrin-dependent, we demonstrate that BACE1 is sorted to early endosomes via an alternative route controlled by the small GTPase ARF6. Indeed, blocking this route with a GTP-locked ARF6-Q67L mutant prevented BACE1, but not APP, from reaching early endosomes. Altering ARF6 levels or activity affects therefore endosomal sorting of BACE1, and consequently results in altered APP processing and Aβ production. Furthermore, sorting of newly internalized BACE1 from ARF6-positive towards RAB5-positive early endosomes depends on its carboxyterminal DISLL motif. In primary hippocampal neurons, endogenous ARF6 essentially localizes to soma and dendrites, underscoring that this ARF6-mediated sorting of BACE1 is confined to the somatodendritic compartment. Using dual-microfluidic chambers, in which axons can be physically separated from dendrites, we could demonstrate that major BACE1-mediated ectodomain shedding and secretion of amyloid β peptides occurs primarily in somatodendritic compartments.

## Conclusion

Taken together, we conclusively demonstrate that BACE1 reaches the early endosomal compartment through a mechanism mediated by ARF6, which is principally distinct from the clathrin-dependent route operated by APP. Our study therefore demonstrates that BACE1 and the APP can be independently sorted by interfering with the expression and/or activity of regulatory proteins in the respective endosomal-sorting machineries. Among these are coat proteins as well as small GTPases and their activating/deactivating components. These findings pave the way for researchers to identify factors or compounds that selectively affect the endocytic sorting and recycling of either BACE1 or APP, allowing for the development of therapies that reduce the production of neurotoxic Aβ in the brain. As such, this study harbors a largely unexplored therapeutic potential for Alzheimer’s disease.

